# Comparison between two devices in the detection of corneal thickness changes after cataract surgery

**DOI:** 10.1038/s41598-021-86158-6

**Published:** 2021-03-23

**Authors:** Maddalena De Bernardo, Livio Vitiello, Giulia Abbinante, Ilaria De Pascale, Luigi Capasso, Giuseppe Marotta, Nicola Rosa

**Affiliations:** 1 Department of Medicine, Surgery and Dentistry, “Scuola Medica Salernitana”, University of Salerno, Via S. Allende, Baronissi, 84081 Salerno, Italy; 2 Corneal Transplant Unit, ASL Napoli 1, Naples, Italy

**Keywords:** Eye diseases, Imaging techniques

## Abstract

This study compared corneal thickness (CT) changes obtained with specular microscopy (SM) and a rotating Scheimpflug camera (RSC) after conventional phacoemulsification surgery (PS). One hundred sixty six eyes of 83 patients were analyzed before and one month after PS. One eye underwent PS, while the fellow phakic one was used as control. CT was measured with SM at the center of the cornea and with RSC at the pupil center, at the corneal apex and at the thinnest point. In the operated eye, SM showed a larger CT mean increase than those one detected at the three different measurements’ points evaluated by RSC. Inversely, in the fellow phakic eye, SM showed a greater CT mean decrease than those one registered by RSC at its three measurement’s points. Thus, one month after surgery, even if cornea appears clear at the slit-lamp, a significant thickness increase is still present. This is even more evident if the slight decrease of the fellow phakic eye is considered. The differences between the two devices are probably related to the different measured areas.

## Introduction

Recently, a precise corneal thickness (CT) evaluation has triggered an ever-increasing interest, as a reliable predictor of corneal health^1^. Furthermore, in clinical practice CT is used in case of corneal refractive surgery^2–6^, to differentiate contact lens-induced corneal warpage from keratoconus^7^, endothelial cells health and function^8–10^, intraocular pressure (IOP) evaluation^11,12^. CT measurements can be achieved with devices based on different techniques such as: confocal microscopy, optical coherence tomography (OCT), slit scanning corneal tomography (Orbscan), optical pachymetry with rotating Scheimpflug camera (Pentacam, Precisio, Galilei), dual beam partial coherence interferometry (PCI), contact and non-contact specular microscopy (SM), ultrasound biomicroscopy (UBM) and ultrasound pachymetry (UP).

Currently, in case of CT measurements, UP is considered the gold standard. The portability of this instrument is an advantage, but the corneal contact requires topical anesthesia^13^, and exposes to the risk of corneal infection and trauma, moreover the position of the probe repeatability is related to the operator’s skill^14–16^.

On the contrary, non-contact pachymetry with specular microscopy or Scheimpflug camera do not require anesthesia, are risk-free for corneal infections or trauma, are less dependent on the operator’s skill and are highly comfortable for the patient.

To our knowledge, CT measurement obtained with Nidek CEM-530 SM and Pentacam HR has never been compared, in patients who underwent cataract surgery.

In a previous study, CT measurements obtained with Pentacam and Nidek CEM-530 in normal subjects were compared and Pentacam measurements were found to be thinner than those obtained with CEM-530^17^. The aim of this study was to check if such differences persisted in patients after uneventful phacoemulsification surgery (PS)^18^, where a slight subclinical oedema could be present, trying to highlight differences in detecting potential changes before and after the surgical procedure.

## Materials and methods

### Patients selection

In this observational case–control study, 83 patients (44 females, mean age: 72.9 ± 8.2 years, range: 51–88 years) who underwent uneventful conventional phacoemulfication were included. Patients with systemic and ocular diseases (e.g. corneal opacities and/or ectasias, keratoconus) and subjects who underwent refractive surgery were excluded. CT measured with both Nidek CEM-530 and Pentacam HR were compared before and one month after the surgical procedure, at the time of lens prescription. For each patient, one eye underwent surgery, while the fellow phakic one was used as control, to check if the difference of readings before and after was due to surgery and not related to the normal measurements’ variation^19^.

The present study adhers to the ethical principles of the Declaration of Helsinki.

All the patients were carefully informed about the purpose of the study and written informed consent from each participant was acquired. Institutional Review Board (IRB) approval was also obtained from the ComEtico Campania Sud (CECS), Prot. No. 16544.

### Instruments and methods

Pentacam HR (Oculus, Wetzlar, Germany, version 1.19r11) utilizes a rotating Scheimpflug camera and a monochromatic slit light source (blue led at 475 nm), rotating together around the optical axes of the eye.

CEM-530 (Nidek Co, Japan) is an optical device that concurrently provides SM and pachymetric measurements.

CT was consecutively and randomly measured by the above-mentioned devices on the same day, between 10 and 12 a.m.

In the present study, measurements were automatically obtained with the two instruments. The patients were instructed to sit in front of the instrument, with their forehead and chin placed on the device supports, holding both eyes open and focusing on a blinking fixation target in the camera’s center. On a computer screen, the operator visualized the image of the patient's eye and centered it by rotating the joystick of the device. When the image was correctly aligned, the patient was advised to remain still, keeping his eyes wide open, so the scan automatically began.

CT values obtained with CEM-530 from central cornea were compared with the three different CT points provided by Pentacam, named pupil center (PC), corneal apex (CA) and thinnest point (TP).

### Statistical analysis

All data were put in a Microsoft Excel spreadsheet and MedCalc 19.5.3 (Mariakerke, Belgium) was used for statistical evaluation. Kolmogorov–Smirnov test showed a normal distribution (*p* > 0.05) for all data. For this reason, Student paired T-test and Pearson correlation coefficient were performed^20,21^. Furthermore, reliability and agreement between the two devices were calculated, using the Bland–Altman plots, intraclass correlation coefficient (ICC), and 95% confidence interval of limits of agreement^22,23^.

*P* values less than 0.05 were considered statistically significant.

Maximizing the statistical power, sample size was determined. To perform the analysis, G*Power software (version 3.1.9.4) was utilized. Sample size was computed with two-tailed significance level (α = 0.05) and 90% power^24^. A difference between two dependent means (matched pairs) was calculated. Effect size was set as medium at around 0.361. Results showed: actual power = 0.902; non-centrality parameter δ = 3.289; critical t = 1.989; Df = 82; total sample size = 83.

## Results

CT measurements with both CEM-530 and Pentacam HR and their respective ICC and Pearson correlation coefficients are summarized in Tables 1 and 2, showing that Nidek results are thicker than the Pentacam ones, both before and after surgery.

**Table 1:** Preoperative and postoperative CT values for operated and fellow eye.

	Nidek CEM-530		Pentacam PC		Pentacam CA		Pentacam TP	
	Preop	Postop	Preop	Postop	Preop	Postop	Preop	Postop
**Operated eye**								
Mean	558	564	546	551	548	552	541	543
SD	32	35	33	34	32	33	32	33
Min	469	479	465	470	466	472	461	465
Max	643	663	632	635	631	638	629	629
R	0.92		0.89		0.89		0.87	
**Fellow eye**								
Mean	560	557	547	546	549	548	541	540
SD	34	31	31	30	31	30	31	31
Min	491	489	479	482	484	489	470	470
Max	646	640	621	618	623	620	613	611
R	0.97		0.96		0.95		0.95	

*PC* pupil center, *CA* corneal apex, *TP* thinnest point, *SD* standard deviation, *R* Pearson correlation coefficient.

**Table 2:** ICC and 95% CI for operated and fellow eye.

	Operated eye				Fellow eye			
	Preoperative		Postoperative		Preoperative		Postoperative	
	ICC	95% CI	ICC	95% CI	ICC	95% CI	ICC	95% CI
Nidek-pentacam PC	0.93	0.90 to 0.96	0.96	0.93 to 0.97	0.95	0.92 to 0.97	0.95	0.92 to 0.97
Nidek-pentacam CA	0.95	0.92 to 0.97	0.96	0.93 to 0.97	0.95	0.93 to 0.97	0.95	0.92 to 0.97
Nidek-pentacam TP	0.92	0.88 to 0.95	0.94	0.91 to 0.96	0.93	0.89 to 0.95	0.94	0.91 to 0.96

*PC* pupil center, *CA* corneal apex, *TP* thinnest point, *ICC* intraclass correlation coefficient, *95% CI* 95% confidence interval.

Concerning the operated eye, with CEM-530 a CT mean increase of 6 ± 14 µm (*p* < 0.05) was found. With Pentacam HR a mean increase of 5 ± 16 µm at the PC (*p* < 0.05), 4 ± 15 µm at the CA (*p* < 0.05), 2 ± 16 µm at the TP (*p* = 0.26) was found, respectively.

In the fellow phakic eye, with CEM-530 a CT mean decrease of  − 3 ± 9 µm (*p* < 0.05) was found. With Pentacam HR a mean decrease of  − 1 ± 9 µm at the PC (*p* = 0.23),  − 1 ± 9 µm at the CA (*p* = 0.19),  − 1 ± 10 µm at the TP (*p* = 0.28).

In addition, focusing on the operated eye and considering the differences between the preoperative and postoperative evaluations with the two devices, a statistically significant difference can be noticed only comparing CEM-530 with Pentacam HR TP (*p* < 0.05), whereas no statistically significant differences were found comparing CEM-530 with Pentacam HR PC and CA (*p* = 0.32 and *p* = 0.09, respectively). In other words, the two instruments in these points (PC and CA) show a similar mean CT difference, comparing the preoperative and postoperative evaluation.

**Figure 1:**
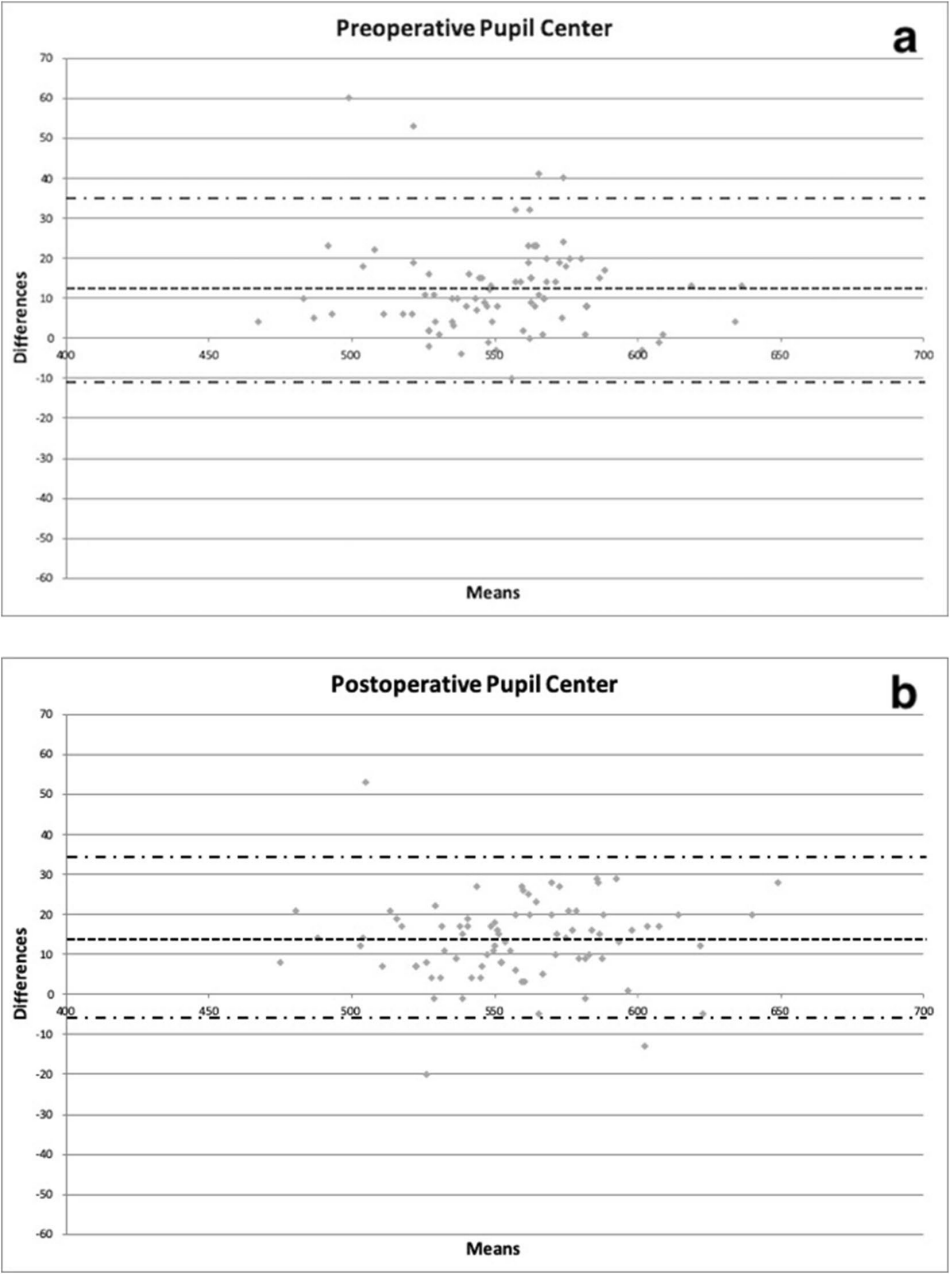
Bland–Altman plots between preoperative (**a**) and postoperative (**b**) corneal thickness in microns obtained with CEM-530 and Pentacam HR at the pupil center. Dashed line: mean difference. Dash-dotted lines: mean ± 1.96 standard deviation of the differences.

**Figure 2:**
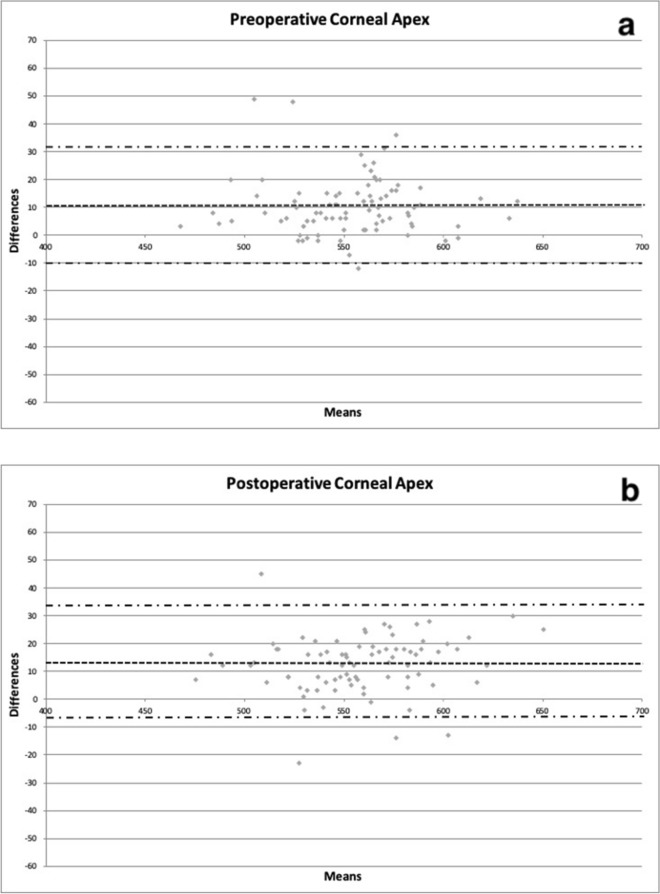
Bland–Altman plots between preoperative (**a**) and postoperative (**b**) corneal thickness in microns obtained with CEM-530 and Pentacam HR at the corneal apex. Dashed line: mean difference. Dash-dotted lines: mean ± 1.96 standard deviation of the differences.

**Figure 3:**
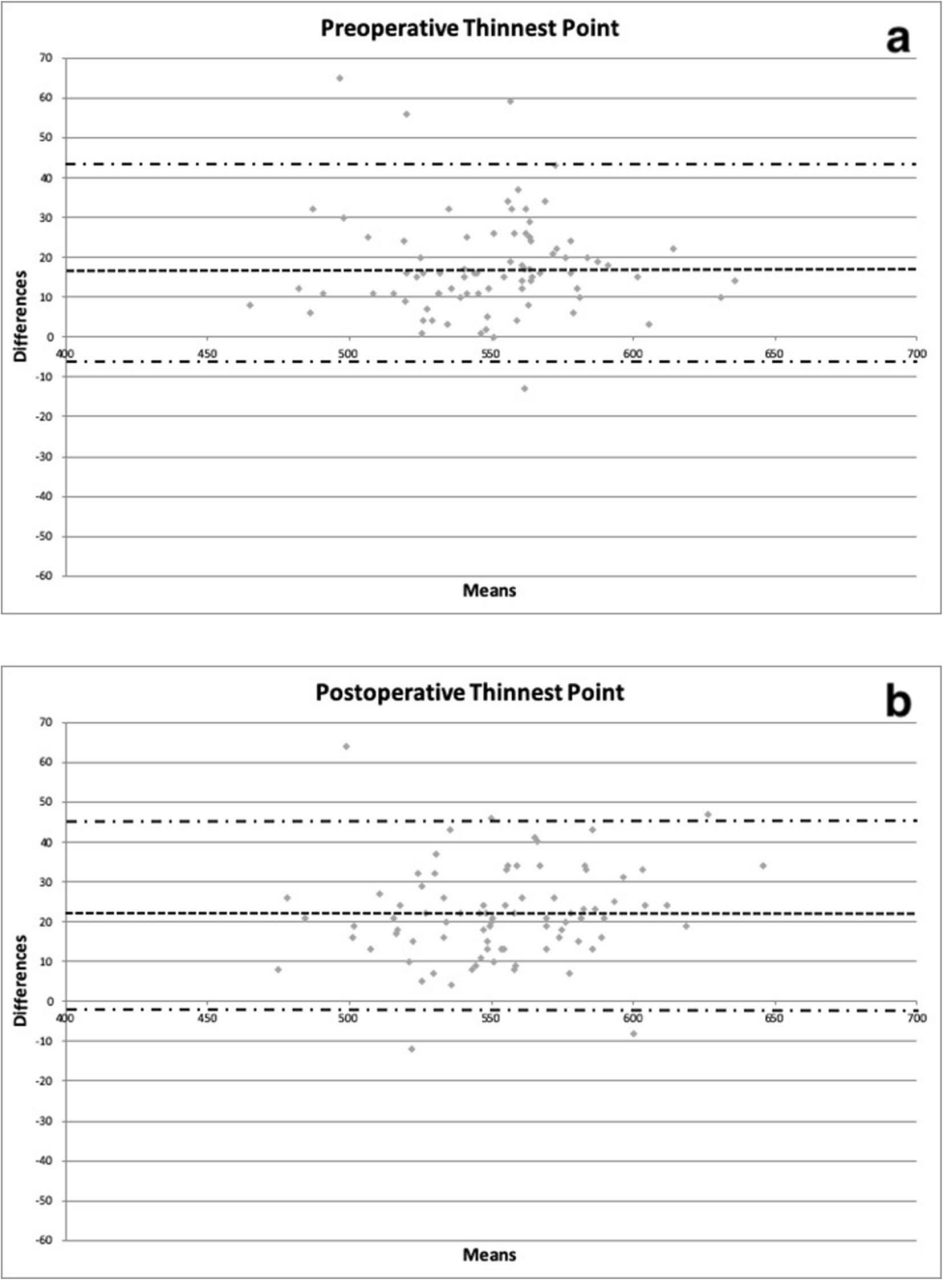
Bland–Altman plots between preoperative (**a**) and postoperative (**b**) corneal thickness in microns obtained with CEM-530 and Pentacam HR at the thinnest point. Dashed line: mean difference. Dash-dotted lines: mean ± 1.96 standard deviation of the differences. GPower: https://www.psychologie.hhu.de/arbeitsgruppen/allgemeine-psychologie-und-arbeitspsychologie/gpower.html.

Pearson correlation coefficients and limits of agreement between preoperative and postoperative data obtained by each device are summarized in Table 3. Bland-Altmann plots are depicted in Figs. 1, 2 and 3.

**Table 3:** 95% Limits of agreement (LoA) preoperative and postoperative operated eye.

	Upper limit	95% CI	Lower limit	95% CI	R
**Preoperative operated eye**					
Nidek-pentacam PC	+ 35	+ 31 to + 40	− 11	− 15 to − 6	0.94
Nidek-pentacam CA	+ 31	+ 27 to + 35	− 10	− 14 to − 6	0.95
Nidek-pentacam TP	+ 43	+ 38 to + 47	− 7	− 12 to − 3	0.92
**Postoperative operated eye**					
Nidek-pentacam PC	+ 34	+ 30 to + 38	− 7	− 11 to − 3	0.96
Nidek-pentacam CA	+ 33	+ 29 to + 36	− 8	− 12 to − 4	0.96
Nidek-pentacam TP	+ 45	+ 41 to + 50	− 2	− 6 to + 3	0.94

*PC* pupil center, *CA* corneal apex, *TP* thinnest point, *R* Pearson correlation coefficient.

## Discussion

Nowdays various devices are available for CT evaluation, therefore agreement studies are essential. Several authors evaluated corneal thickness changes in eyes subjected to cataract surgery, but most of them evaluated a small number of patients. Moreover, as far as we can tell, a comparison between these two devices before and after PS has never been performed.

In a study carried out with optical low coherence reflectometry and ultrasonic pachymetry on 30 patients, Ventura et al.^25^ showed a considerable corneal thickness increase the day after surgery, that returned to the preoperative values in a period ranging from three months to one year.

In another study conducted with an optical low-coherence reflectometer slit lamp pachymeter on 13 patients, Salvi et al.^26^ found a CT return to the preoperative value from the first week.

Kandarakis et al.^27^ showed the same trend in 41 patients, but with values still high at seven days compared to the preoperative ones.

Utilizing Orbscan II topographer on 124 patients, De Juan^28^ detected a significant decrease in CT that lasted up to the second week after cataract surgery, the period in which it tends to stabilize. On the other hand, the same authors noticed less significant CT decreases from the second to the fourth week when it usually tends towards the starting values. Caglar et al.^29^ and Calabuig-Goena et al.^30^ seem to have confirmed, in more recent times, these results in their studies.

Dzhaber et al.^31^, comparing CT measurements, provided by a non-contact SM, in 67 patients who underwent both conventional phacoemulsification surgery and femtosecond laser-assisted cataract surgery, found a non significant CT increase in both groups one month after surgery.

Concerning the comparison between instruments in normal patients that did not undergo cataract surgery, only two studies, utilizing the same devices used in the current research, were carried out by Karaca et al.^32^ on 100 eyes of 50 healthy volunteers and by De Bernardo et al.^17^ on 209 right eyes of 209 healthy patients.

Karaca et al.^32^ assessed CT values with Oculus Pentacam, Nidek CEM-530, and Konan Medical CellChek XL. They concluded that there is a good correlation between these three devices, with significant differences between CEM-530 and Pentacam (*p* < 0.01). In particular, they reported CEM-530 measurements to be thinner than those obtained with Pentacam.

On the contrary, De Bernardo et al.^17^ found a good correlation between CEM-530 and Pentacam HR, with CEM-530 producing a thicker corneal measurement than Pentacam HR.

Compared to the aforesaid studies, in the present study, a group of operated eyes matched to the fellow phakic ones was evaluated. The same protocol was also used in the study of Salvi et al.^26^, but unfortunately, only a limited number of patients was evaluated.

Furthermore, to our knowledge, the present study compared for the first time CEM-530 and Pentacam HR CT values in patients before and after PS.

In the operated eye, from these results, it is possible to highlight an increase in CT after one month. This is more evident with CEM-530 (*p* < 0.05), but it is also shown by Pentacam HR at PC (*p* < 0.05) and CA (*p* < 0.05), whereas no significant difference was found at TP (*p* = 0.15).

In our opinion, this statistically significant difference could be related to the unstable TP location, while PC and CA are stable.

In fact, the corneal thinnest point is not the same during repeated measurements over time but tends to change along with corneal remodeling following PS.

This hypothesis could be strengthened when the two devices' differences between preoperative and postoperative period are considered, as a statistically significant difference, in this case, can be noticed just at the TP.

On the other hand, in the fellow phakic eye, no significant differences exist between the first and second Pentacam HR examination time, while CEM-530 points out a decrease in CT measurement (*p* < 0.05). This is probably related to the less accuracy of CEM-530 compared to Pentacam HR. Some of the reasons could be the lack of exam quality check provided by the instrument and the different areas evaluated by the two devices.

Moreover, both in operated and fellow phakic eye, CEM-530 supplies thicker CT values than Pentacam HR. These results, contrarily to Karaca et al.^32^, agree with the results provided by De Bernardo et al.^17^. Probably, this could be explained by the different devices utilized in these studies: Pentacam HR in the present and in the previous study by De Bernardo et al., and Pentacam in the Karaca et al. study^32^, though it could appear less plausible.

Another explanation could be related to the different points in comparison with the ones analyzed by Karaca et al.^32^, as well as Ucakhan et al.^33^. In fact, contrary to these studies, they did not report which RSC corneal parameters were compared with those provided by SM.

We acknowledge that this study has some limitations, among them, the potentially different area measured by the two devices, the short-term follow-up period and the lack of the endothelial cell count, as an increase in CT could be related to a decrease in cell count. To better evaluate the long term PS effects on CT, further studies with close-up measurements over time and a longer follow up should be performed.

In conclusion, although a great correlation between CEM-530 and Pentacam HR was found, these two devices should not be interchangeably utilized, as shown by Bland-Altmann plots.

In addition, one month after surgery, even if cornea appears clear at the slit-lamp, a significant corneal thickness increase is still present. This is even more evident if the slight decrease of the fellow phakic eye is considered. The differences obtained with the two devices are probably related to the different area measured by the two devices.

## Data Availability

The datasets generated and analyzed during the current study are available from the corresponding author on reasonable request.
